# Arabidopsis *CULLIN3* Genes Regulate Primary Root Growth and Patterning by Ethylene-Dependent and -Independent Mechanisms

**DOI:** 10.1371/journal.pgen.1000328

**Published:** 2009-01-09

**Authors:** Alexis Thomann, Esther Lechner, Maureen Hansen, Eva Dumbliauskas, Yves Parmentier, Joe Kieber, Ben Scheres, Pascal Genschik

**Affiliations:** 1 ZMBP–Developmental Genetics, Universität Tübingen, Tübingen, Germany; 2 Institut de Biologie Moléculaire des Plantes, Centre National de la Recherche Scientifique, Unité Propre de Recherche 2357, Conventionné avec l'Université Louis Pasteur, Strasbourg, France; 3 Department of Biology, University of North Carolina, Chapel Hill, North Carolina, United States of America; 4 Molecular Genetics, Department of Biology, Utrecht University, Utrecht, The Netherlands; Iowa State University, United States of America

## Abstract

CULLIN3 (CUL3) together with BTB-domain proteins form a class of Cullin-RING ubiquitin ligases (called CRL3s) that control the rapid and selective degradation of important regulatory proteins in all eukaryotes. Here, we report that in the model plant *Arabidopsis thaliana*, *CUL3* regulates plant growth and development, not only during embryogenesis but also at post-embryonic stages. First, we show that CUL3 modulates the emission of ethylene, a gaseous plant hormone that is an important growth regulator. A CUL3 hypomorphic mutant accumulates ACS5, the rate-limiting enzyme in ethylene biosynthesis and as a consequence exhibits a constitutive ethylene response. Second, we provide evidence that *CUL3* regulates primary root growth by a novel ethylene-dependant pathway. In particular, we show that *CUL3* knockdown inhibits primary root growth by reducing root meristem size and cell number. This phenotype is suppressed by ethylene-insensitive or resistant mutations. Finally, we identify a function of *CUL3* in distal root patterning, by a mechanism that is independent of ethylene. Thus, our work highlights that *CUL3* is essential for the normal division and organisation of the root stem cell niche and columella root cap cells.

## Author Summary

Ubiquitin-mediated proteolysis plays a central role in controlling intracellular levels of essential regulatory molecules in all eukaryotic organisms. This protein degradation pathway has a large number of components, including hundreds of ubiquitin protein ligases (E3s) that are predicted to have regulatory roles in cell homeostasis, cell cycle control, and development. Recent research revealed the molecular composition of CULLIN3 (CUL3)-based E3 ligases, which are essential enzymes in both metazoans and plants. Here, we report that in the model plant *A. thaliana*, *CUL3* modulates the emission of ethylene, a gaseous plant hormone that controls a variety of processes such as fruit ripening and stress response. In particular, we provide evidence that *CUL3* regulates root growth by a novel ethylene-dependant pathway. Thus, we showed that *CUL3* knockdown inhibits primary root growth by reducing the root meristem size. Finally, we also identified a function of *CUL3* in distal root patterning. Indeed, *CUL3* function is required for normal division and organisation of the root stem cell niche and columella root cap cells. Overall, our results show that Arabidopsis *CUL3* is essential for plant growth and development, not only during embryogenesis but also at post-embryonic stages.

## Introduction

Regulation of protein stability through the ubiquitin proteasome system (UPS) is now considered as a major mechanism underlying many cellular and organismal processes, such as cell division, DNA repair, quality control of newly produced proteins, developmental and immune defense pathways, and in plants, light and phytohormone signal transduction [1]–[3]. Degradation *via* the UPS is a two-step process: the protein is first tagged by covalent attachment of ubiquitin and subsequently degraded by a multicatalytic protease complex called the 26S proteasome. The transfer of ubiquitin to a target protein substrate requires an ubiquitin protein-ligase (E3).

E3 enzymes act to specify the substrates and thus they play a key role in the ubiquitylation reaction. Several hundred different E3s have been identified in metazoan and plant genomes, based on specific, commonly shared structural motifs. However, the most intensively studied subclasses of E3s are those of the cullin-RING ligase (CRL) superfamily, which form multi-protein complexes [4]. CRL E3s can be viewed as two functional modules brought together by the CULLIN proteins, acting as molecular scaffolds. The first module forms the catalytic centre and is composed of a RING finger domain protein and an ubiquitin conjugating enzyme (E2). The second module can be considered as the substrate recognition module, in which a specific protein physically interacts with the target substrate.

A series of recent reports has shed light on the molecular composition and function of the CUL3-based CRL E3s (reviewed in [5]). Certain ‘Bric a brac, Tramtrack and Broad Complex/Pox virus and Zinc finger’ (BTB/POZ) domain proteins function as substrate specific receptors in *Schizosaccharomyces pombe* and *Caenorhabditis elegans* [6]–[9]. These BTB domain proteins bind CUL3, via the BTB domain and direct substrate specificity through an independent protein-protein interaction domain. The best-documented substrate for the CUL3-BTB ligases thus far, is the nematode MEI-1 protein, which regulates the meiosis-to-mitosis transition of fertilized embryos [6], [8]–[9]. In mammals, *CUL3* function is essential and its loss-of-function in mouse produces an arrest during early embryogenesis [10]. Recent data have also implicated vertebrate CUL3 in cell cycle regulation [11] and signal transduction pathways, such as the Wnt-beta-catenin pathway [12]. In contrast to metazoans, the function of the *CUL3* orthologs is not essential in budding and fission yeasts [7],[13].

The Arabidopsis genome encodes two CUL3-related proteins, called CUL3A and CUL3B [14]. Disruption of both genes causes embryo lethality [15]–[17], indicating that CRL3s play important functions during early steps of plant development. Moreover a genomic search revealed the existence of about 80 BTB-domain proteins in *Arabidopsis* belonging to different families depending on additional protein domains, either upstream or downstream of the BTB-domain, such as the meprin and TRAF homology (MATH) domain, the Armadillo repeats (ARM) and the tetratricopeptide repeats (TPR) [15]–[16], [18]–[19]. Protein interaction studies in yeast suggested that CUL3A and CUL3B may form many different CRL3 complexes, but their nature and substrates are still poorly documented in plants.

ETO1, an *Arabidopsis* BTB-domain protein, controls the stability of ACS5, a member of the 1-aminocyclo-propane-1-carboxylic acid synthases (ACS) that catalyse a rate-limiting step in ethylene biosynthesis [20]. Moreover, ETO1 was found to directly interact with CUL3A, which prompted the authors to propose ACS5 as the first reported substrate for a plant CUL3-based E3. However ETO1 is also able to inhibit ACS5 activity without CUL3, indicating a higher complexity in this regulation [20]. Supporting a function of CRLs in ethylene biosynthesis is the fact that cycles of (de)neddylation seem to play an important role in this process, because Arabidopsis RNAi lines in which the expression of two NEDD8-related proteins, RUB1 and RUB2, are reduced exhibit a triple response and overproduce ethylene [21].

To better characterize the function of CRL3 E3s in Arabidopsis, we identified a hypomorphic mutation in *CUL3A*, which, when combined with the *cul3b* null mutation strongly impairs overall CRL3 functions. Here we report a molecular and genetic characterization of this line, with a focus on ethylene biosynthesis and primary root growth.

## Results

### Generation of a Hypomorphic Mutant Reveals Different *CUL3* Functions during Arabidopsis Development

**Figure 1:**
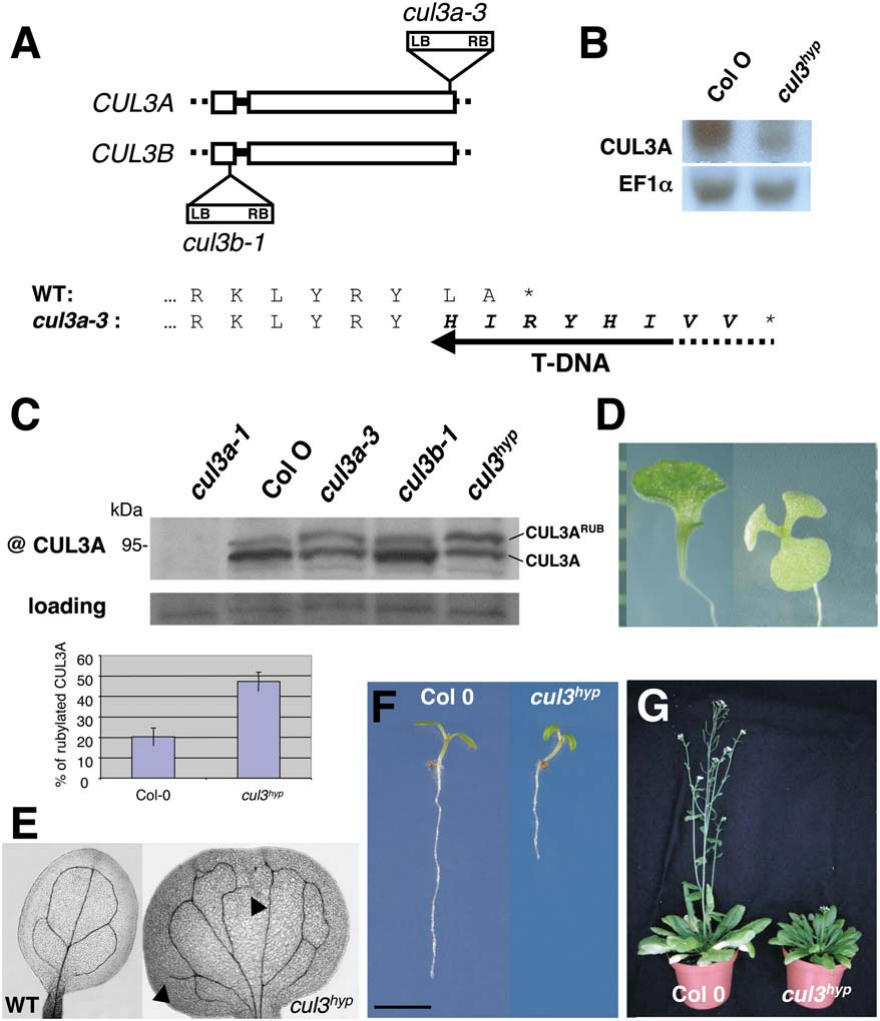
The *cul3^hyp^* displays multiple developmental abnormalities. A. Schematic representation of T-DNA insertions in both *CUL3A* and *CUL3B* genes. LB and RB indicate the orientation of the left and right T-DNA borders, respectively. Exons (open boxes), introns (single lines) and 5′ and 3′UTRs (dots) are indicated. Below, C-terminal protein sequence of CUL3A aligned with the *cul3a-3* mutant protein. The extra eight-residue peptide is indicated in bold. B. *CUL3A* transcript level in wild type and mutant plant. RNA was extracted from 3-week-old plants. C. CUL3A protein detected by a CUL3A-specific anti-peptide antibody. Proteins were extracted from 3-week-old wild-type and mutant plants as indicated. Rubylated and unrubylated CUL3A proteins are indicated. The panel below shows the percentage of rubylated CUL3A protein in wild type and *cul3^hyp^*. Data are means of 3 different western blots ±SE. D. 10-day old *cul3^hyp^* mutant seedlings showing cotyledon phenotype. E. Venation defects of *cul3^hyp^* cotyledon. F. 4-day-old light grown wild type and *cul3^hyp^* mutant seedlings. G. 58-day-old wild type and *cul3^hyp^* mutant plants under short days conditions.

Previously it was shown that the combined disruption of both Arabidopsis *CUL3A* and *CUL3B* genes causes embryo lethality [15]–[17]. To further investigate the function of *CUL3* in plants, we searched for additional Arabidopsis T-DNA insertion lines. One line was of particular interest as the T-DNA was inserted at the very end of the *CUL3A* coding sequence. This mutant allele, called *cul3a-3* was further characterised. The T-DNA insertion creates a mutation, in which the last two amino acids of the protein are replaced by an eight-residue peptide (Figure 1A). The *cul3a-3* mutant line produces a lower abundant truncated transcript (Figure 1B). Interestingly, the CUL3A protein detected by a specific anti-peptide antibody [18] was not only less abundant, but was also hyper-rubylated (Figure 1C), suggesting that the truncated CUL3A protein is less prone to de-rubylation. It is noteworthy that cycles of rubylation/de-rubylation are important for CRLs activity (reviewed in [22]).

Homozygous *cul3a-3* mutant plants are fertile and do not show morphological defects under normal growth conditions. As *CUL3A* and *CUL3B* genes are functionally redundant, we generated a double mutant using the previously characterized *cul3b-1* knockout line [17]. The double homozygous *cul3a-3 cul3b-1* mutant, hereafter called *cul3* hypomorph (*cul3^hyp^*), was viable, but exhibited several developmental defects. Approximately 10% of homozygous *cul3^hyp^* seedlings displayed altered cotyledon phenotypes (Table 1). Some seedlings exhibited a single cotyledon while others, at a lower frequency, had three cotyledons (Figure 1D); seedlings with partially or totally fused cotyledons were also observed (not shown). In addition, the vascular patterning of cotyledons was often abnormal (Figure 1E). In particular, we observed interrupted and freely ending veins. In less than 1% of these seedlings we observed other abnormalities, such as root-less seedlings (not shown). The absence of root meristem was previously revealed in some of the Arabidopsis *cul3a-1 cul3b-1* double null mutants that could complete their embryonic development [17].

**Table 1:** Scoring of the cotyledon phenotype observed with *cul3^hyp^* seedlings.

Alles	Dicotyledons	Monocotyledon	Tricotyledons	Fused cotyledons	Seedlings scored
Wild type	1010	0	0	0	1010
*cul3^hyp^*	2637 (89.25%)	225 (7.61%)	64 (2.16%)	29 (0.98%)	2955

The subset of *cul3^hyp^* seedlings that had normal organ patterning displayed epinastically curled cotyledons and shorter roots when grown in the light (Figure 1F). At a latter stage of development, the most striking phenotype was a reduced rosette size and a delay in flowering (Figure 1G). Consistently, a slight delay in flowering was observed in single *cul3a-1* loss-of-function mutant [18].

Overall, our data indicate that Arabidopsis *CUL3A* and *CUL3B* are important for plant growth and development, both during embryogenesis and at post-embryonic stages.

### The *cul3^hyp^* Double Mutant Displays a Triple Response in the Absence of Ethylene

**Figure 2:**
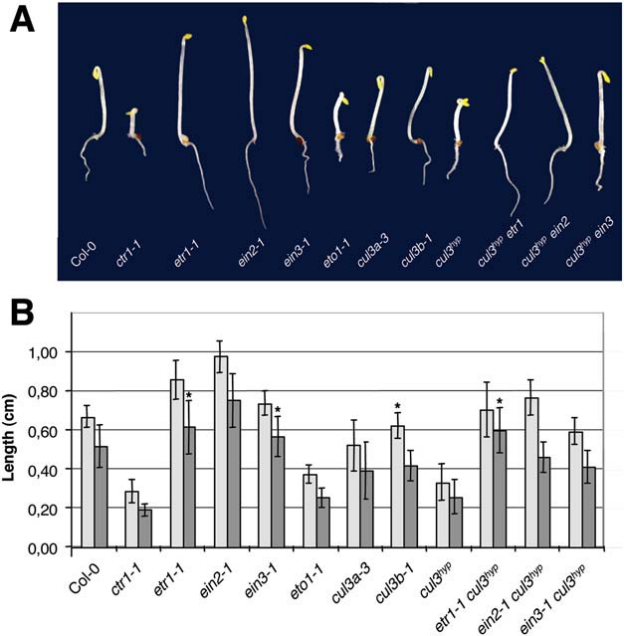
The *cul3^hyp^* mutant exhibits the triple response in the dark. A. Phenotypes of 3-day-old etiolated seedlings of the indicated genotypes grown without ACC. B. Hypocotyl (light grey) and root (dark grey) length measurements of 3-day-old etiolated seedlings of the indicated genotypes grown without ACC. Values are average lengths (means±SD) of >30 hypocotyls or roots. T-tests were performed for each value compared to WT (the triple mutants were compared to their corresponding ethylene-insensitive mutants) indicating significant differences (p<0.05). The (*) symbol highlights values for which p>0.05.

Because ETO1 is involved in the ethylene biosynthetic pathway and physically interacts with Arabidopsis CUL3A [20], most likely through its BTB domain, we investigated whether the *cul3^hyp^* mutant is affected in ethylene-mediated processes. In accordance with this speculation, etiolated *cul3^hyp^* mutants displayed a typical triple response in the absence of ethylene, which is characterized by short hypocotyls, short roots, and exaggerated apical hooks (Figure 2). The phenotype was similar to that of *eto1-1*, though less severe than the *constitutive triple response1* (*ctr1-1*) mutant. It is noteworthy that the single *cul3a-3* mutant displayed a weak triple response. Moreover, when germinated in the presence 5 µM ACC, the *cul3^hyp^* mutant was still responsive to ethylene in a root elongation assay (Figure S1).

To better characterize whether the *CUL3A* and *CUL3B* genes are involved in the control of ethylene biosynthesis or might also play functions further downstream in the signalling cascade, we first used a pharmacological approach. We found that treatment with 2 µM aminoethoxyvinyl glycine (AVG), which inhibits ACC synthase and hence ethylene biosynthesis [23], significantly reversed the *cul3^hyp^* triple response (Figure S1). However, AVG has toxic effects and it inhibited root elongation of wild-type plants even at low concentrations (not shown).

Thus, we undertook a genetic approach and generated triple mutant combinations with ethylene-insensitive or resistant mutants in the ethylene-signalling cascade. In all triple mutants, *etr1-1 cul3^hyp^*, *ein2-1 cul3^hyp^* and *ein3-1 cul3^hyp^*, the triple response observed in the *cul3^hyp^* hypomorph was significantly, but not entirely suppressed (Figure 2). We conclude that the triple response phenotype of *cul3^hyp^* can be mostly explained by a function of *CUL3A* and *CUL3B* upstream of ethylene perception. However, the fact that both hypocotyl and root length were slightly reduced in all three triple mutants compared to their corresponding single ethylene insensitive or resistant mutants indicates that Arabidopsis *CUL3* genes act also at other levels, which are ethylene independent.

### Ethylene Production of cul3^hyp^ Is Significantly Enhanced by the *eto1-1* and *etr1-1* Mutations

We generated an *eto1-1 cul3^hyp^* triple mutant and found only a slight additive effect on the triple response regarding root growth (Figure S1). These data indicate that the triple response observed in *cul3^hyp^* is mainly attributed to a defect in the CUL3^ETO1^ E3 ligase, but does not exclude the possibility that the two ETO1-related proteins, EOL1 and/or EOL2 [20] may also play some minor roles in this process.

**Figure 3:**
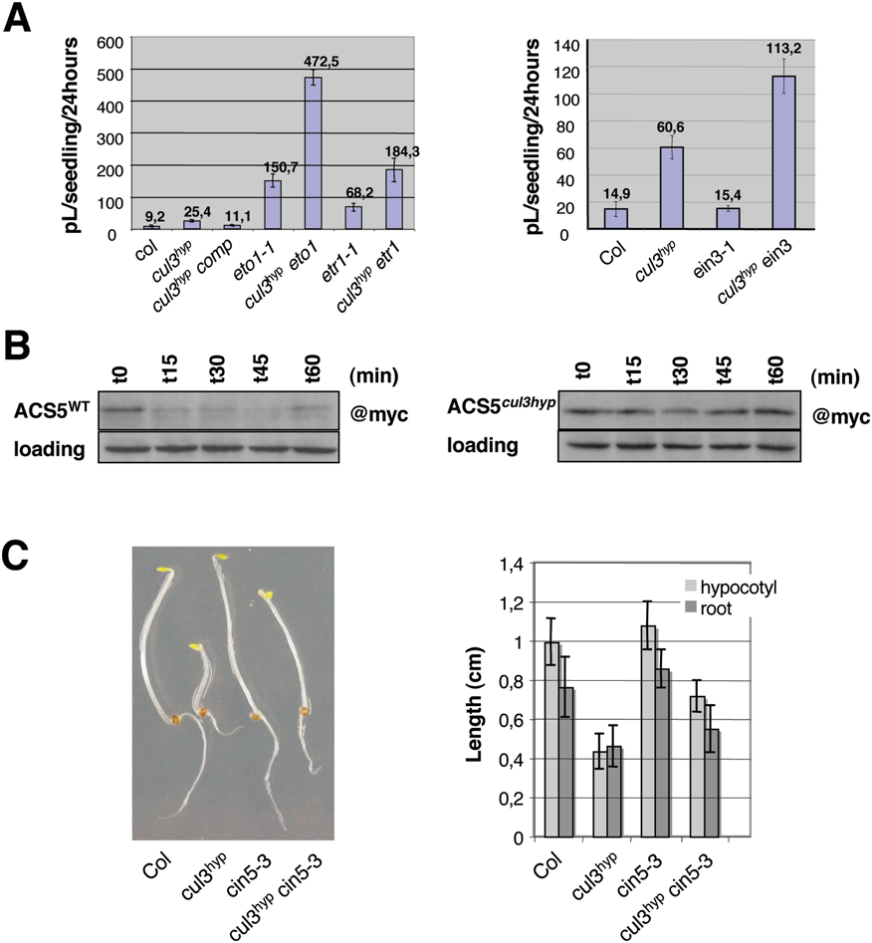
Ethylene production and ACS5 turnover in the cul3^hyp^ mutant. A. Ethylene emission from combinatorial mutants of the indicated genotypes. Etiolated seedlings were grown in sealed vials and the amount of ethylene released was quantified by gas chromatography (pL, pico Liter). Each bar represents the average (±SE) of three separate vials with each vial measured in triplicate. B. The half-life of myc-ACS5 is increased in *cul3^hyp^* mutant seedlings. Wild type (left panel) or *cul3^hyp^* mutant (right panel) seedlings harbouring the myc-ACS5 transgene were grown for 4 days on MS medium containing either 1 nM DEX (wild type) or 10 nM DEX (*cul3^hyp^*) in the dark. The seedlings were washed in liquid MS medium lacking DEX and then suspended in liquid MS medium lacking DEX but containing the protein synthesis inhibitor cycloheximide at time 0. At various times (indicated in minutes above each lane), the seedlings were harvested, and protein extracts were analyzed by immunoblotting using an anti-myc monoclonal antibody. C. Phenotypes of 3-day-old etiolated seedlings of the indicated genotypes grown without ACC (left panel). Hypocotyl (light gray) and root (dark gray) length measurements of 3-day-old etiolated seedlings of the indicated genotypes grown without ACC (right panel). Values are average lengths (means±SD) of >30 hypocotyls or roots. For all mutants (except *cin5-3*) p<0.05 compared to wild type plants.

As a next step, we measured ethylene emission between day 3 and day 4 in the etiolated mutant seedlings. Consistently, the *cul3^hyp^* mutant accumulated two to three-fold more ethylene gas than did the wild type control (Figure 3A), but significantly less than *eto1* seedlings. To further confirm that the triple response observed in the etiolated seedlings is the consequence of *CUL3A/B* knockdown, we transformed the *cul3^hyp^* double mutant with a binary vector spanning a *CUL3A* genomic fragment [18]. Several transformants were recovered, which suppressed the triple response (not shown) as well as ethylene overproduction (Figure 3A).

Because the triple response of the *eto1-1 cul3^hyp^* triple mutant was slightly more severe than in the *eto1-1* single mutant (Figure S1), we measured ethylene production in the triple *eto1-1 cul3^hyp^* mutant (Figure 3A). There was an increase of about 3-fold in ethylene production as compared to the *eto1-1* parent, suggesting that CUL3A/B controls ethylene production by both ETO1-dependent and independent mechanisms.

There was also an increase of about 3-fold in ethylene production in the *etr1-1 cul3^hyp^* mutant compared to *etr1-1* (Figure 3A). Likewise, ethylene production was increased in the *ein3-1 cul3^hyp^* triple mutant compared to the *cul3^hyp^*parent, despite the fact that *ein3-1* does not overproduce ethylene. This data indicates that CUL3A/B acts additively with the autoinhibition control of ethylene biosynthesis.

### ACS5 Is a Target of CUL3 in Arabidopsis

ETO1 directly interacts with ACS5 to inhibit its activity, but also mediates ACS5 26S proteasome-dependent degradation, most likely via CUL3A/B [20]. Moreover, the *cin5-3* mutation, which disrupts the *ACS5* gene [24], significantly reduces ethylene production in *eto1-1* and partially suppresses its constitutive triple response [25]. Thus, we speculated that the constitutive triple response observed in *CUL3A/B* knockdown was mainly the consequence of ACS5 protein stabilisation. To address this issue, we first introgressed a transgenic line expressing a Dex-inducible myc-tagged ACS5 [25] into the *cul3^hyp^* double mutant background. Due to partial silencing of the *ACS5* reporter construct in the *cul3* hypomorph, we could not compare directly the myc-ACS5 protein accumulation in *cul3^hyp^* and wild-type backgrounds at identical concentrations of dexamethasone (Dex). However, by increasing Dex levels, we could normalize the expression of myc-ACS5 in *cul3^hyp^* and compare myc-tagged ACS5 protein half-lives in both genetic backgrounds (Figure 3B). After Dex-induction and subsequent removal, seedlings were incubated in presence of cycloheximide, which blocks *de novo* protein synthesis, and the myc-tagged ACS5 protein levels were then determined by immunoblot analysis. Whereas ACS5 protein in the wild type background had a very short half-life of ±15 min as previously reported [25], there was no decrease in the level of ACS5 protein after 1 hour. Thus, Arabidopsis CUL3A and CUL3B are involved in the turnover of the ACS5 isoform. Furthermore, we produced the *cin5-3 cul3^hyp^* triple mutant and found that *cin5-3* suppresses partially the triple response of *cul3^hyp^* (Figure 3C). Overall, we conclude that ACS5 is a primary target of CUL3A/B and ETO1 in seedlings, but other ACSs, in particular of the type-2 class, are most likely also degraded by this E3 ligase.

### *CUL3A* and *CUL3B* Regulate Primary Root Growth by a Mechanism Different from *ETO1*

The *cul3^hyp^* mutant exhibits a shorter root (Figure 1F) and *CUL3A/B* genes are essential during embryogenesis for proper patterning of the hypophyseal lineage, important founders of the future root meristem [17]. Furthermore, the role of ethylene on primary root growth was recently emphasized by several reports [26]–[29]. To characterize the function of CUL3A/B during root development, the work focused on primary root growth.

**Figure 4:**
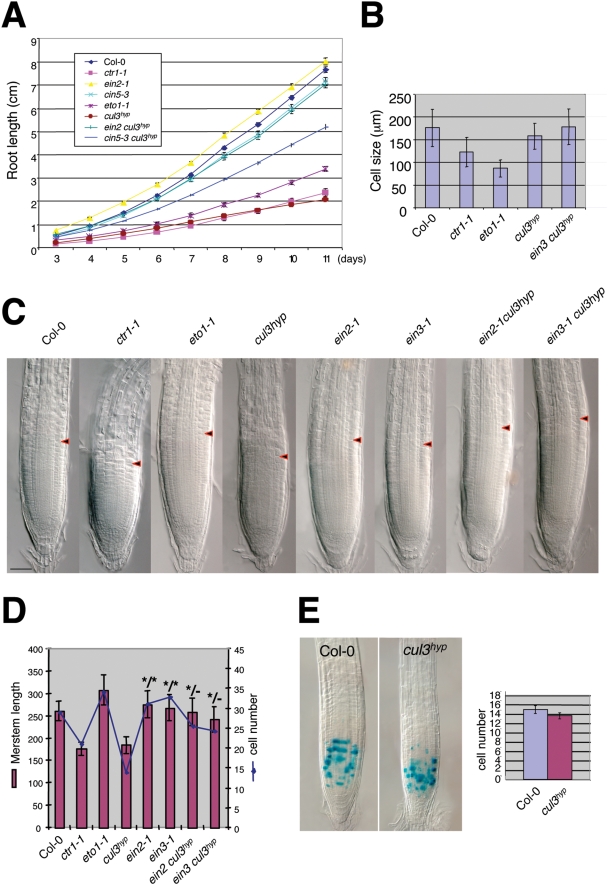
The *cul3^hyp^* mutant affects primary root meristem size and cell number. A. Kinetic analysis of primary root elongation of wild type compared to the indicated genotypes. Values are average lengths of >16 roots. B. Cortical cell lengths in the differentiation zone of 7-day-old wild type and mutant roots. Values are average lengths (means±SD) of more than 60 cells measured in 5 different roots. C. Representative wild type and mutant root tips. Arrows indicate the transition zone where cells leave the meristem and enter the differentiation zone. D. Root-meristem size indicated in µm and cell number of wild-type plants compare to the indicated genotypes. Cortex meristematic cells showing no sign of differentiation were counted. Values are average lengths or numbers (means±SD) of >12 roots at 7 dpg. T-tests were performed for each value compared to WT (the triple mutants were compared to their corresponding ethylene-insensitive mutants) indicating significant differences (p<0.05). The (*) symbol highlights values (meristem size/cell number) for which p>0.05. E. Mitotic activity in wild type and *cul3^hyp^* mutant root meristems, which is monitored by the proCYCB1;1-GUS reporter. Values in the right panel are average numbers of stained cells (means±SE) of more than 18 roots.

At 11 days post-germination, the elongation of *cul3^hyp^* primary root showed a reduction of about 80% as compared to wild type (Figure 4A and Figure S2). The root growth defect was similar to *ctr1*. *ein2-1* and *ein3-1* (not shown) significantly suppressed this phenotype, but these mutations were unable to restore wild type root growth. Thus, *CUL3A* and *CUL3B* regulate primary root growth via both ethylene-dependent and independent mechanisms.

To better characterise this phenotype, we measured the length of cells in the cortex in the differentiation zone, because it was found that ethylene stimulates auxin biosynthesis and its basipetal transport to the root elongation zone, where auxin inhibits cell elongation [27],[29]. Consistent with such a scenario, we observed a 50% reduction in length of these cells in *eto1-1* (Figure 4B). However to our surprise, cortical cell length in the differentiation zone was only marginally affected in the *cul3^hyp^* mutant. This suggests that the mechanism by which ethylene inhibits root growth in a *CUL3*-deficient mutant background is different from that reported for *eto1* mutants and ACC-treated wild-type plants [27],[29].

Root growth depends on cell elongation and on cell production rates in the root apical meristem. Therefore we investigated whether *CUL3A/B* knockdown affects the meristem size and/or activity. Strikingly, we observed that in *cul3^hyp^* the meristem size and cell number was reduced compared to wild-type plants (Figure 4C–D). Interestingly, in the *ctr1-1* mutant, there was also a significant reduction in both meristem size and cell number. Conversely, in *eto1-1*, the root meristem size and cell number were even slightly increased in comparison to wild-type plants, suggesting that ethylene overproduction in this mutant can have opposite effects in the root, negatively affecting cell expansion in the elongation zone but positively affecting root meristem size.

Importantly, the meristem phenotype of *cul3^hyp^* was suppressed by the *ein2-1* and *ein3-1* mutations and therefore is dependent on ethylene signalling. We investigated whether this phenotype in *cul3^hyp^* was the consequence of reduced cell cycle activity. To this end we introduced the p_CYCB1;1_::GUS reporter construct [30] into *cul3^hyp^* mutant background. The Destruction box of plant B-type mitotic cyclins targets proteins for degradation after mitosis [31] and thus the p_CYCB1;1_::GUS reporter is a suitable marker to identify cells in G2-to-early M phase. The number of CycB;1:GUS expressing cells was not significantly reduced in the root apical meristem of *cul3^hyp^* compared to wild-type plants (Figure 4E). Thus, the most likely explanation of the ethylene-dependent inhibition of root growth is that cells in the *cul3^hyp^* mutant prematurely exit the meristem and make the transition to cell expansion.

### The *cul3^hyp^* Mutation Affects Distal Root Patterning in an Ethylene-Independent Manner

**Figure 5:**
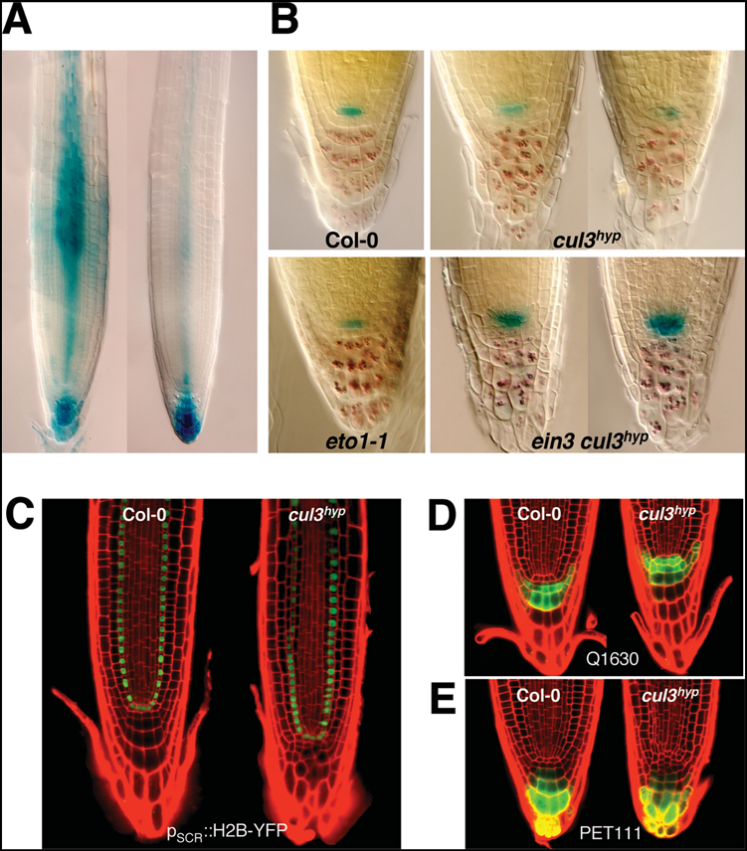
The *cul3^hyp^* mutant shows defects in QC and columella cells organisation. A. Expression patterns of p_CUL3A_::GUS and p_CUL3B_::GUS in primary roots. B. Double labelling of QC and columella cells in wild type and different mutants. The QC46 marker was used to visualize GUS staining (in blue) in the functional QC. Lugol staining was used to visualize the differentiated columella cells. C. Expression of the endodermis and QC marker pSCR::H2BYFP in wild type (left) and *cul3^hyp^* mutant (right) roots. D. and E. Columella-specific enhancer trap Q1630 and PET111 in wild type (left) and cul3^hyp^ mutant (right) roots. Q1630 is only expressed in columella layers C1 and C2.

To get more insights into the mechanism(s) by with *CUL3A/B* regulate root growth, we determined the expression pattern of both genes in Arabidopsis roots by using promoter-GUS fusions. After short GUS staining, we observed the strongest histochemical p_CUL3A_::GUS localization in the stele, but also in the distal part of the root (Figure 5A). A similar expression pattern was also observed for the p_CUL3B_::GUS reporter, although the signal was weaker in the stele. In the *cul3^hyp^* mutant, this region revealed clear defects of cell division patterns in the quiescent center (QC), other cells of the root stem cell niche and the columella root cap (Figure 5B–E). Starch granule staining, which marks only differentiated columella cells, showed premature differentiation of the columella root cap initials (Figure 5B). To investigate whether this defect in root meristem patterning is associated with mis-specification of the QC or columella cells, we examined the expression of different markers in the *cul3^hyp^* mutant background. In *cul3^hyp^* plants expressing QC-specific marker QC46 (Figure 5B) or endodermis and QC marker p_SCR_::H2BYFP (Figure 5C), we could identify signal in cells of the QC region, even when their morphology or cell number was altered. However, when we used columella-specific markers (Figure 5D–E) and in particular Q1630, which is only expressed in layers C1 and C2 in wild type (n = 21), we observed a different pattern in the *cul3^hyp^* mutant plants. The marker was in general expressed in additional columella cell layers (50%, n = 32) or showed a patchy distribution (22%, n = 32).

It was recently reported that ethylene modulates cell division in the QC, which can eventually lead to additional columella cell layers [26]. Therefore we investigated whether the phenotype observed in *cul3^hyp^* is dependent on ethylene signalling. However, this was not the case as defects of cell division in the QC and columella remained in the *ein3-1 cul3^hyp^* triple mutant (Figure 5B). Moreover, contrarily to the report of Ortega-Martinez et al. [26], we did not observe deregulated QC cell divisions in the *eto1-1* mutant. Thus, we conclude that the knockdown of *CUL3A/B* function in Arabidopsis disturbs distal root patterning by a mechanism that is ethylene independent.

### PIN7 Protein Accumulates in cul3^hyp^ Collumella Cells by a Post-Transcriptional Mechanism

**Figure 6:**
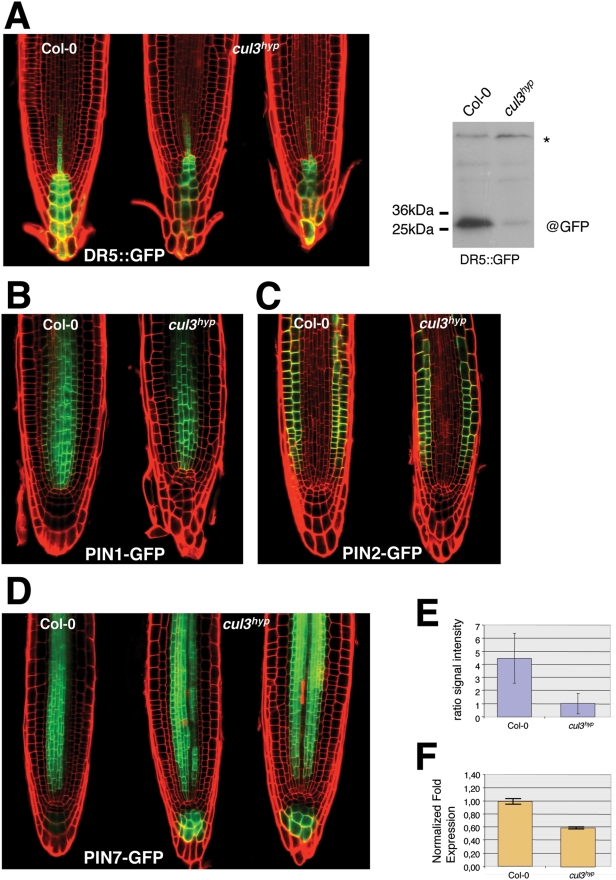
The *cul3^hyp^* mutant accumulates PIN7 in the root cap. A. On the left panel, expression of the auxin-sensitive reporter DR5rev-GFP in wild type (left) and *cul3^hyp^* mutant (middle and right) roots. On the right panel, immuno-detection of GFP protein from wild-type and *cul3^hyp^* mutant root tips expressing the auxin-sensitive reporter DR5rev-GFP. The asterisk indicates an aspecific protein band used as loading control. B. PIN1 expression and polarity monitored by the p_PIN1_::PIN1-GFP reporter in wild type (left) and *cul3^hyp^* mutant (right) roots. C. PIN2 expression and polarity monitored by the p_PIN2_::PIN2-GFP reporter in wild type (left) and *cul3^hyp^* mutant (right) roots. D. PIN7 expression and polarity monitored by the p_PIN7_::PIN7-GFP reporter in wild type (left) and *cul3^hyp^* mutant (middle and right) roots. E. Ratio of the GFP signal between the stele and the columella tissues. F. Relative levels of *PIN7* transcripts (determined by real-time RT-PCR) in 10 day-old root tips (1 cm of the root starting from the root tip) in *cul3^hyp^* and Col control plants. Data are means±SE.

Auxin is involved in distal pattern formation of Arabidopsis roots [32]. To investigate whether auxin signalling is affected in *cul3^hyp^*, we introduced the DR5rev-GFP reporter construct into the *cul3^hyp^* mutant and monitored GFP expression in the root tip (Figure 6A). The spatial distribution of DR5 expression in the mutant was more narrow and also reduced in intensity in comparison to wild type roots (Figure 6A), indicating that auxin signalling is reduced in the distal part of *cul3^hyp^* roots.

**Figure 7:**
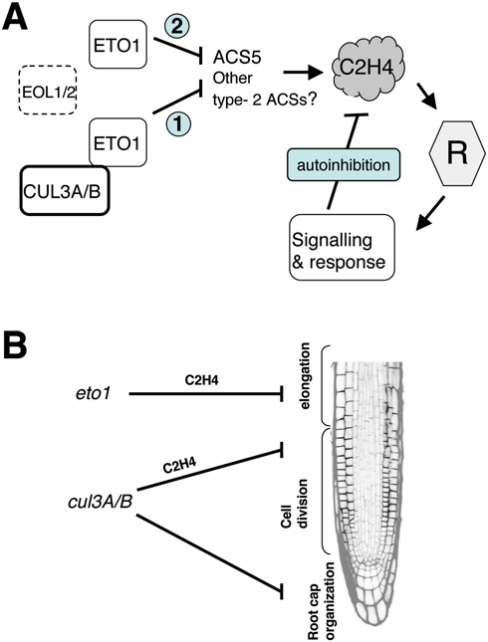
Models for CUL3A/B action in ethylene biosynthesis and root growth. A. Proposed model based on our genetic interactions to explain the role of CUL3A and CUL3B in ethylene biosynthesis. ETO1-dependent and independent effects on ethylene emission have been observed. (1) CUL3A/B^ETO1^ controls ACS5 and most likely other type-2 ACSs protein stability. (2) ETO1 inhibits ACSs also independently of CUL3A/B [20]. The negative feedback mechanism from ethylene perception and signal transduction to ethylene biosynthesis is also shown. B. We propose a model in which the ethylene pathway acts at two different levels to inhibit primary root growth. The first mode of action triggered by ethylene is an inhibition of cell elongation as earlier reported [27],[29]. This mechanism most likely involves ETO1 and can be mimicked by exogenous application of ethylene. The second mechanism affects cell differentiation at the exit of the meristem and involves *CUL3A/B*.

PIN-FORMED (PIN) proteins are rate-limiting factors catalysing polar auxin transport [33]–[34]. These proteins are crucial for auxin distribution and as such provide positional information to coordinate plant development. Because auxin signalling was reduced in the *cul3^hyp^* distal part of the root, one possibility is that the auxin gradient is disturbed in this mutant. Thus, we introduced into the *cul3^hyp^* mutant background different *PIN::PIN-GFP* reporters, consisting of their endogenous promoters and translational fusions between PIN1, PIN2 and PIN7 proteins and GFP. Whereas PIN1 and PIN2 expression patterns and levels were similar to wild type in *cul3^hyp^* (Figure 6B–C), we observed a higher expression level of PIN7 in columella cells (Figure 6D). The ratio of the GFP signal between the stele and the columella tissues was four-to-five times higher in *cul3^hyp^* roots in comparison to wild type (Figure 6E). *PIN* gene expression is regulated at the transcriptional, but also post-transcriptional levels [35]. Thus we performed quantitative RT-PCR assays on *PIN7* gene expression on isolated wild type and *cul3^hyp^* root tips. In contrast to the PIN7 protein accumulation in columella cells, the PIN7 transcript level in *cul3^hyp^* was slightly reduced in comparison to wild type (Figure 7F). Our data indicate that *CUL3A/B* knockdown induces in the highest CUL3 expression domain PIN7 accumulation, most likely by a post-transcriptional mechanism.

## Discussion

### The *cul3^hyp^* Mutant Controls Ethylene Production

ACSs are rate-limiting enzymes in ethylene biosynthesis (reviewed in [36]). Arabidopsis has nine ACSs, which are subdivided into three different types [37]. ETO1, a BTB domain-containing protein interacts with type-2 ACS proteins and mediates the degradation of at least one of them, ACS5 [20]. Because ETO1 also interacts in a yeast two-hybrid assay with Arabidopsis CUL3A, it was concluded that ACS5 becomes ubiquitylated by a CUL3^ETO1^ E3 ligase and subsequently degraded by the 26S proteasome [20],[37].

Our data indicate that ethylene production is induced in the *cul3^hyp^* mutant, which is consistent with such a scenario. In addition, we found that the half-life of ACS5 is prolonged in the *cul3^hyp^* mutant providing a molecular evidence for the involvement of CUL3A/B in the turnover of ACS5. However it is noteworthy that *cul3^hyp^* is a weak ethylene overproducer in comparison to *eto1-1*, *cul3^hyp^* produces about six-fold less ethylene than does *eto1-1*. Thus, it is possible that this difference in ethylene production is the consequence of a longer ACS5 half-life in *eto1* in comparison to *cul3^hyp^* mutant. This is also consistent with the fact that *cul3^hyp^* still keeps some CRL3 activity, whereas *eto1-1* is a null allele. However, we also cannot rule out that ETO1, which is still present in the *cul3^hyp^* mutant background, inhibits ACS5 protein activity by a process independent of protein degradation. Moreover, BTB-containing proteins themselves are also substrates for these CUL3 complexes [8],[38], which may even lead to a higher ETO1 protein accumulation in the *cul3^hyp^* mutant. This data would be consistent with the finding that ETO1 overexpression in Arabidopsis reduces kinetin-induced ethylene production and most importantly that ETO1 inhibits ACS5 activity via a direct interaction [20].

Arabidopsis ETO1 is part of a small gene subfamily containing two other closely related BTB-domain proteins, called EOL1 and EOL2 [20]. Genetic interactions showed only minor additive effects on the triple response morphology in *eto1-1 cul3^hyp^* triple mutant compared to *eto1-1*, but a strong increase in ethylene production was observed. This suggests that EOL1 and EOL2 are also involved in ethylene biosynthesis, though ETO1 is the main CUL3 receptor in this process. Although it was previously shown that the single *eol1* and *eol2* mutants grow like wild type in the dark [16], recent data demonstrated that both EOL1 and EOL2 negatively regulate ethylene biosynthesis by directing type-2 ACS proteins for degradation [39].

Is ACS5 the only ACS target of CUL3A/B in Arabidopsis? Based on our data we can say that ACS5 is a primary target, because ACS5 loss-of-function in *cin5-3* significantly suppressed the triple response during early seedling development (Figure 3C) and the root growth inhibition (Figure 4A) of *cul3^hyp^*. Nevertheless, *cin5-3* does not entirely revert the *cul3^hyp^* mutant; thus, it is likely that other ACSs are also targeted by CUL3A/B^ETO1^ (Figure 7A). As for ETO1, EOL1 and EOL2 only interact with type-2 ACSs [37],[39],whereas type-1 and type-3 ACSs, which are also degraded by the 26S proteasome [40]–[41], are most likely recognized by another Arabidopsis E3 ligase.

It was previously found that ethylene-insensitive or resistant mutants, such as *etr1-1*, *ein2-1* and *ein4-1* produce increased amounts of ethylene [42]–[44], whereas *ctr1-1* does not [45]. This suggested a negative feedback mechanism from ethylene perception and signal transduction to ethylene biosynthesis [43]. The finding that the *cul3^hyp^* mutation synergistically enhances ethylene production in *etr1-1* indicates that *CUL3A/B* and *ETR1* act in parallel and most likely independently in the regulation of ethylene biosynthesis (Figure 7A). This is consistent with previous studies showing that ETO1 also acts synergistically with *etr1* to enhance ethylene production [46].

### Ethylene Inhibits Primary Root Growth by Two Different Mechanisms

Arabidopsis primary root growth is reduced in a concentration-dependent manner when plants are exposed to ACC or to exogenously applied ethylene and this process is the consequence of down regulation of cell elongation ([47] and references therein). Recent results demonstrated that ACC treatment of wild-type plants positively regulates auxin biosynthesis and distribution in Arabidopsis roots [27]–[29]. Based on different approaches, a model was proposed in which ethylene-stimulated auxin is subsequently basipetally transported to the elongation zone where it inhibits cell elongation [27],[29].

In the present report we provide evidence that the ethylene pathway also acts on root development at a different level (Figure 7B). We show that the inhibition of *CUL3A/B* activity impairs primary root growth in an ethylene-dependent manner by reduction of root meristem size and cell number. In contrast to *eto1-1* or ACC-treated plants [27],[29],[47], no significant effect was observed on cell elongation in the *cul3^hyp^* mutant. However, we found a similar root phenotype in *ctr1-1* and the double *ebf1-1 ebf2-1* [48] mutant backgrounds (this work and data not shown), both accumulating the transcription factor EIN3 leading to a constitutive ethylene response. It is noteworthy that the *ctr1-1* mutation affects both cell elongation and root meristem size, indicating that ethylene signalling is involved in both mechanisms. Because the number of mitotic cells in the *cul3^hyp^* root meristem was not significantly different from wild type, we conclude that the reduced meristem size is the consequence of the premature transition of cells from the meristem to the cell elongation zone, rather than caused by differences in cell division activity.

An intriguing observation is that in *eto1-1*, which overproduces ethylene, or in ACC-treated roots ([27] and our data), no decrease on the root meristem size was observed. This suggests that despite the fact that ethylene is a volatile gas, depending on its sites of production and/or perception, ethylene can induce different local responses. Hence, this fits with the evidence that ACS genes in Arabidopsis display distinct expression patterns during plant development [49].

### *CUL3A/B* Activity Is Necessary for Normal Root Cap Organisation

The strong *CUL3A* and *CUL3B* expression in the distal part of the root and their involvement in distal root patterning suggest that CUL3A/B proteins control the division and organisation of the stem cell niche and columella root cap cells. By which mechanism(s) do *CUL3A/B* genes maintain QC and root cap organisation? It was recently reported that ethylene modulates cell division in the stem cell niche [26]. Such an effect of ethylene would be consistent with the role of *CUL3A/B* in ethylene biosynthesis (see above). However, several lines of evidence argue against this possibility. First, 1 µM ACC treatment of wild type roots revealed no abnormalities in columella cell differentiation and/or tissue organisation [27]. Second, such a phenotype was not observed in the *eto1-1* null mutant. Third and most importantly, the ethylene insensitive mutation *ein3-1* did not suppress cell division defects in the QC and in columella cells. A possible explanation for the discrepancies between our data and [26] is that the two *eto1-11* and *eto1-12* alleles used in their studies, which are point mutations, are acting in a dominant negative way to inhibit CUL3A/B activity. Overall, even if a subtle effect of ethylene on stem cell division is not excluded, our data point to an ethylene-independent role of CUL3A/B in organizing stem cell niche and columella (Figure 7B).

Interestingly, we noticed that the DR5::GFP signal in the *cul3^hyp^* root tip was weaker and more restricted than in wild type roots, suggesting altered auxin signalling. In addition, we also observed a higher PIN7 protein accumulation in *cul3^hyp^* columella cells. One way to connect these observations is a scenario in which PIN7 missexpression leads to the depletion of intracellular auxin from the root tip, resulting in reduced auxin signalling and as a consequence patterning defects. Further experiments need to confirm such a model. Moreover, because PIN7 accumulation in columella cells is under post-transcriptional control and because *CUL3A/B* genes are strongly expressed in these cells, it will be interesting to address whether the PIN7 protein is a direct target of a still unknown CRL3 complex. Finally, a broader connection between CUL3A/B and auxin may exist, since several developmental abnormalities in the *cul3^hyp^* mutant, such as altered number of cotyledons, or defects in root and venation patterning and embryogenesis (this work and [17]) are reminiscent of auxin transport or signalling defects.

## Materials and Methods

### Plant Material, Growth Conditions, and Treatment

The Arabidopsis *cul3a-3* (SALK 012973) mutant line has been identified using the web assisted program: http://signal.salk.edu/cgi-bin/tdnaexpress. The insertion site was confirmed by sequencing the T-DNA flanking srquences. The precise location of the T-DNA in the *cul3a-3* mutant has been determined by sequencing, showing an insertion after nucleotide 2191 in the last exon of *CUL3A*. The last 8 nucleotides (TAGCCTAA) of *CUL3A* are replaced by 26 nucleotides from the left border of the T-DNA (ACATACGGTATCATATTGTGGTGTAA) leading to the addition of 8 amino acids (HIRYHIVV) to CUL3A protein sequence.

All *Arabidopsis thaliana* lines used are in the Columbia background except for the Q1630 reporter line, which is in the C24 background. The transgenic and mutant lines have been described elsewhere: *ein3-1* [50], *etr1-1* [51], *eto1-1* [43], *ctr1-1* [45], *ein2-1* [43], *cin5-3* [24], proPIN1-PIN1-GFP and proPIN2-PIN2:GFP [52], proPIN7-PIN7:GFP [53], CYCB1-GUSDB [54], Q1630 [55] and PET111 [56]. The CUL3A- and CUL3B-promoter GUS transgenic lines are described respectively in [18],[19].

*Arabidopsis thaliana* seeds were sterilized with chloral gas or with ethanol method, plated on 1/2 MS medium (1/2 MS salts [Gibco-BRL, Cleveland, OH], pH 5.8, 1% sucrose, and 0.8% agar), stored 2 to 3 days at 4°C in the dark, and then transferred to a plant growth room (21/25°C, 16-h photoperiod). For the triple response assay, surface-sterilized seeds were germinated in the dark on 1/2 MS medium. Plates with seeds were cold-treated at 4°C for 2 to 3 days, exposed to light at room temperature for 2 to 4 h to improve germination, then wrapped with aluminum foil and incubated at 22°C for 3 days in the dark. A minimum of 15 seedlings were scored per mutant by pulling them out of the growth medium, stretching them flat on the surface of another agar plate, taking in pictures, and then quantifying root and hypocotyl lengths using ImageJ (National Institutes of Health; http://rsb.info.nih.gov/ij). For seeds propagation, plants were grown to maturity at 22°C under 16-h photoperiod.

### Transcript Level Analyses

For Northern–blot analysis RNA was extracted from plant material using the Trizol reagent (Invitrogen, Paisley, UK). RNA gel blot analysis was performed with 20 µg of total RNA per lane. Northern blot procedure is described in [31]. ^32^P-labelled probes were synthesized with the Prime-a-Gene random prime labelling kit (Promega Corporation, Madison, WI, 8USA) using a 900-bp CUL3A cDNA fragment amplified with two gene-specific primers (c3as4 5′-ATGGATTTGGGTGAATCTGT-3′ and c3aS5 5′-CTCGGGGTGACTGCCATA-3′).

For qRT-PCR assays, RNA was extracted from 10 days old root tips (1cm of the root starting from the root tip) using the kit NucleoSpin RNA XS (Macherey Nagel). 1 µg of total RNA were reverse transcribed with High Capacity cDNA Reverse Transcription kit TM (Applied Biosystems). PCR was performed using gene-specific primers in a total volume of 15 µL SYBR Green Master mix (Roche) on a Lightcycler LC480 apparatus (Roche) according to the manufacturer's instructions. The TIP4l and At4g26410 genes were used as internal controls. The relative expression level of *PIN7* gene in cul3^hyp^ plants was compared with Col-0 control plants using GenEx Pro 4.3.5. software (MultiD Analyses) after normalization using the At4G26410 cDNA level and averaging over three replicates.

Primer list.

PIN7:TGGGCTCTTGTTGCTTTCA and TCACCCAAACTGAACATTGC

TIP4l: GTGAAAACTGTTGGAGAGAAGCAA and CAACTGGATACCCTTTCGCA

AT4G26410: GAGCTGAAGTGGCTTCAATGAC and GGTCCGACATACCCATGATCC

### myc-ACS5 Protein Stability

Transgenic lines used were dex-inducible myc-tagged ACS5 [25]. The coding region of ACS5 was amplified from cDNA of wild type and fused to a 6× myc cassette, and cloned into the binary GVG vector pTA7002 [57]. Plants were transformed with the plasmids by the floral dip method [58], and transformants were selected on MS medium containing hygromycin. T2 seedlings were grown on MS medium containing 10 nM dex and screened for lines that expressed the myc-tagged proteins at low levels in an inducible manner.

Tissues for protein analyses were ground in a denaturing buffer [59] followed by boiling for 5 min. After centrifugation, 20 µg of total protein extracts were fractionated on a 10% SDS-PAGE gel and blotted onto Immobilon-P membrane (Millipore, Bedford, MA, USA). The immunoreactive proteins were detected using peroxidase-conjugated goat anti-rabbit antibodies (Dianova, Chalfont St Giles, Bucks, UK) and ECL Western blot analysis system from Amersham.

Immunoblots were performed using a 5000-fold dilution of anti-Myc monoclonal antibodies from mouse (clone MYC-1A1 Euromedex) and a 5000-fold dilution of peroxidase-conjugated goat anti-mouse IgG (Molecular Probes). Signals were detected by film (within the linear range of detection) using the enhanced chemiluminescence protein gel blot analysis system (Amersham Biosciences). The blot was stained subsequently with Comassie blue to control the loading.

For ACS5 turnover assays, wild type and *cul3^hyp^* transgenic seedlings harbouring DEX-inducible myc-ACS5 were grown on MS medium for 8 days at 22°C. Twenty ml of liquid MS medium containing different DEX concentrations (see Figure 3) were poured onto the plates where seedlings were growing and incubated for 4 hours. Seedlings were incubated for different times in 1M cycloheximide after three washes of liquid MS medium. Total proteins were extracted and used for immunoblot analysis.

### Ethylene GC Measurements

Seeds were sterilized using chlorine gas sterilization (100 mL bleach +3 mL HCl for ∼4 hours), and seeds were aliquoted in 0.4% top agar to 3 mL MS 1% sucrose media in 22-mL gas chromotography vials. Vials were maintained sterile using autoclaved aluminum foil, for 5 days in 4°C. Vials were incubated in the light for 4–6 hours, then capped and incubated at 22°C dark for 4 days. The vials were capped in the dark at day 3 and incubated to day 4. The accumulated ethylene was measured by gas chromatography as described in [24]. All genotypes are represented by 3 repetitions of 2–3 vials each. Ethylene measured in each vial was then divided by number of seedlings in the vial.

### Phenotypical Analysis and Microscopy

Root seedlings were photographed and their lengths were measured with ImageJ. At least 15 seedlings were processed, and at least three independent experiments were performed, giving the same statistically significant results. Root meristem lengths and epidermal cell lengths were measured on root mounted in chloral hydrate. Images were captured with a Zeiss Axioskop microscope (Carl Zeiss, New York, NY) equipped with a Nikon DXM1200 digital camera (Nikon Instruments Europe, Badhoevedorp, The Netherlands). The number of root meristematic cells was obtained by counting cortical cells showing no sign of vacuolisation. Root meristem length was assessed as the distance between the quiescent center and the first cell with a vacuole. ImageJ was used also for measurements of the length of root cortical cells. At least 10 seedlings were processed in at least three independent experiments giving similar results.

Histochemical GUS staining analyses of the CUL3A/B promoter, the CYCB-GUS and QC reporter lines were done as described in [60]. For the confocal microscopy, roots were visualized using a Leica (Wetzlar, Germany) MZ FLIII fluorescence stereomicroscope equipped with GFP and YFP filters. Propidium iodide (10 µg/mL in distilled water) was used to stain the cell walls of living root cells (red signal). For quantification of PIN7:GFP signal fluorescence ImageJ program was used. The ratio of GFP signal intensity between the stele and the columella was calculated for the wild type and cul3^hyp^ roots. Approximately 15 seedlings/images were examined, in three independent experiments giving similar results.

## Supporting Information

Figure S1 Response of *cul3^hyp^* to AVG and ACC. A. Hypocotyl length measurements of 3-day-old etiolated seedlings of the indicated genotypes grown without (light grey) or with (dark grey) 2 µM AVG. Values are average lengths (means±SD) of >30 hypocotyls. B. Hypocotyl length measurements of 3-day-old etiolated seedlings of the indicated genotypes grown without (light grey) or with (dark grey) 5 µM ACC. Values are average lengths (means±SD) of >30 hypocotyls. C. Root length measurements of 3-day-old etiolated seedlings of the indicated genotypes grown without (light grey) or with (dark grey) 5 µM ACC. Values are average lengths (means±SD) of >30 roots.
(1.5 MB TIF)

Figure S2 Representative 11-day-old seedlings of the indicated genotypes.
(2.8 MB TIF)
