# Supplementary figures and images for: Arabidopsis CULLIN3 Genes Regulate Primary Root Growth and Patterning by Ethylene-Dependent and -Independent Mechanisms
Source: PLoS Genet. 2009 Jan 9;5(1):e1000328. doi: 10.1371/journal.pgen.1000328 (PMC2607017; doi:10.1371/journal.pgen.1000328)

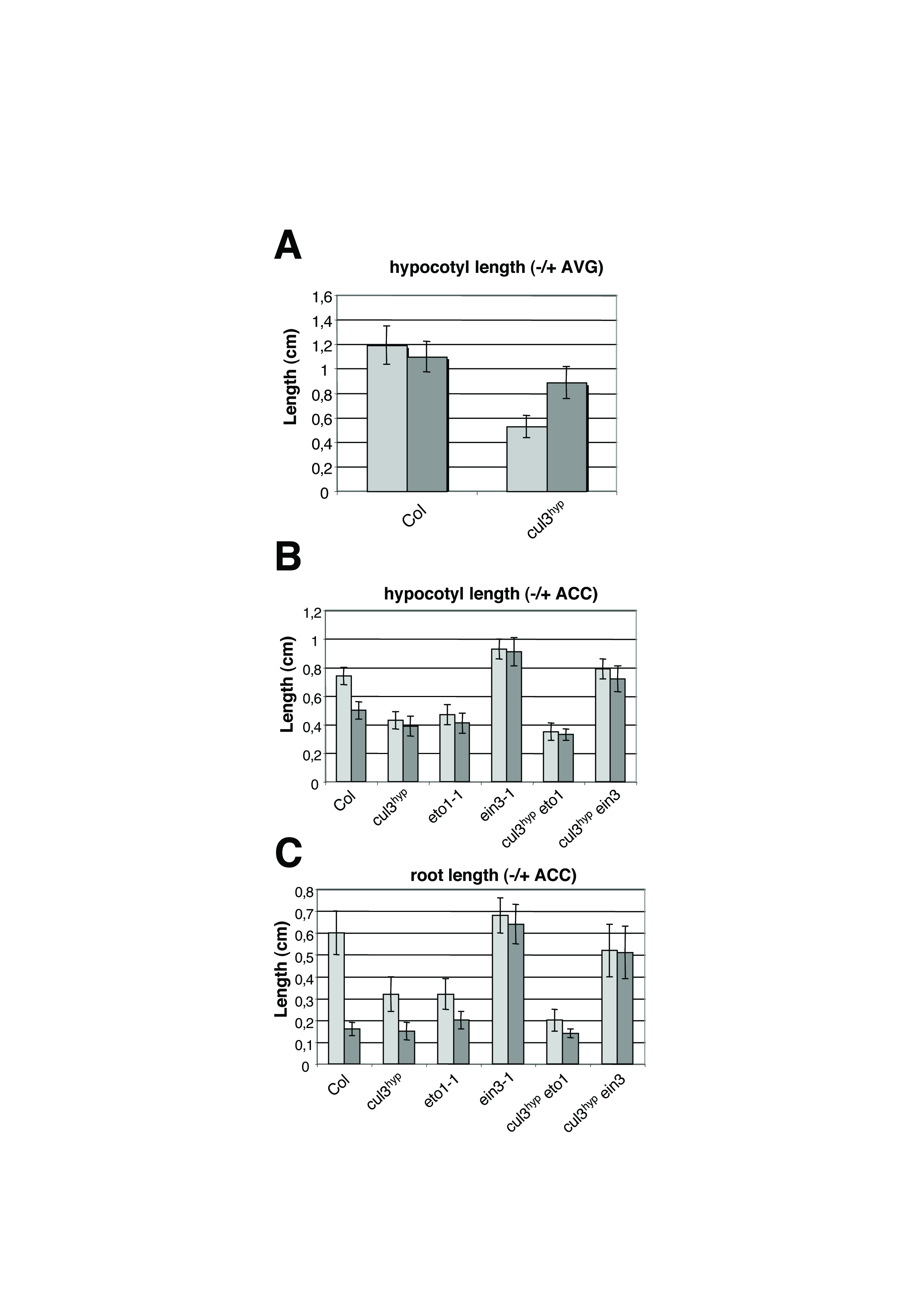

Supplement: Figure S1 — Response of cul3hyp to AVG and ACC. A. Hypocotyl length measurements of 3-day-old etiolated seedlings of the indicated genotypes grown without (light grey) or with (dark grey) 2 µM AVG. Values are average lengths (means±SD) of >30 hypocotyls. B. Hypocotyl length measurements of 3-day-old etiolated seedlings of the indicated genotypes grown without (light grey) or with (dark grey) 5 µM ACC. Values are average lengths (means±SD) of >30 hypocotyls. C. Root length measurements of 3-day-old etiolated seedlings of the indicated genotypes grown without (light grey) or with (dark grey) 5 µM ACC. Values are average lengths (means±SD) of >30 roots. (1.5 MB TIF) [file pgen.1000328.s001.tif]

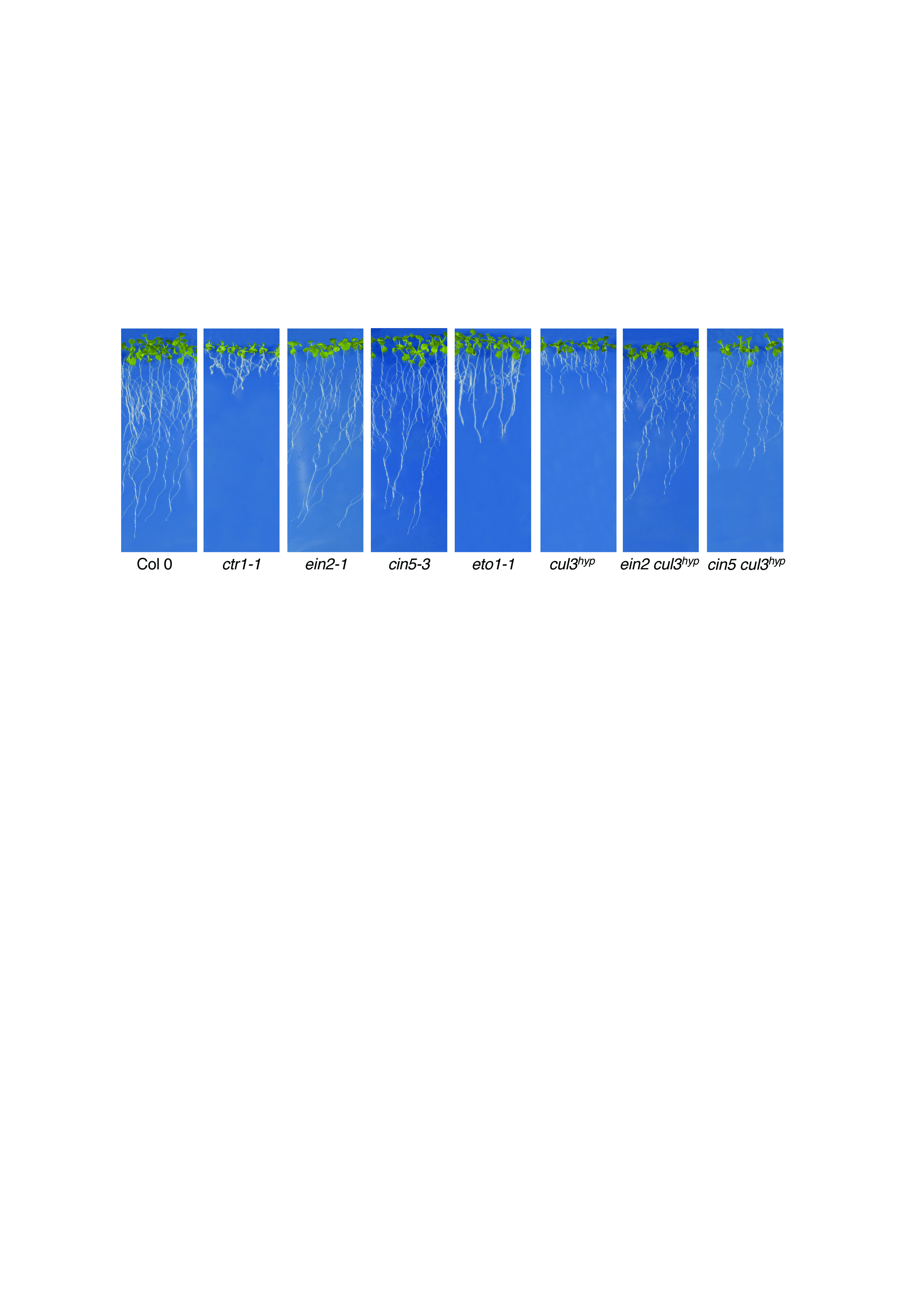

Supplement: Figure S2 — Representative 11-day-old seedlings of the indicated genotypes. (2.8 MB TIF) [file pgen.1000328.s002.tif]
